# Enhancement of Detection of Diabetic Retinopathy Using Harris Hawks Optimization with Deep Learning Model

**DOI:** 10.1155/2022/8512469

**Published:** 2022-05-26

**Authors:** Nagaraja Gundluru, Dharmendra Singh Rajput, Kuruva Lakshmanna, Rajesh Kaluri, Mohammad Shorfuzzaman, Mueen Uddin, Mohammad Arifin Rahman Khan

**Affiliations:** ^1^School of Computer Science and Engineering, VIT, Vellore, India; ^2^School of Information Technology and Engineering, VIT, Vellore, India; ^3^Department of Computer Science, College of Computers and Information Technology, Taif University, Taif 21944, Saudi Arabia; ^4^School of Digital Science, University Brunei Darussalam, Gadong BE1410, Brunei Darussalam; ^5^Department of Computer Science and Engineering, Bangladesh University, Dhaka 1207, Bangladesh

## Abstract

In today's world, diabetic retinopathy is a very severe health issue, which is affecting many humans of different age groups. Due to the high levels of blood sugar, the minuscule blood vessels in the retina may get damaged in no time and further may lead to retinal detachment and even sometimes lead to glaucoma blindness. If diabetic retinopathy can be diagnosed at the early stages, then many of the affected people will not be losing their vision and also human lives can be saved. Several machine learning and deep learning methods have been applied on the available data sets of diabetic retinopathy, but they were unable to provide the better results in terms of accuracy in preprocessing and optimizing the classification and feature extraction process. To overcome the issues like feature extraction and optimization in the existing systems, we have considered the Diabetic Retinopathy Debrecen Data Set from the UCI machine learning repository and designed a deep learning model with principal component analysis (PCA) for dimensionality reduction, and to extract the most important features, Harris hawks optimization algorithm is used further to optimize the classification and feature extraction process. The results shown by the deep learning model with respect to specificity, precision, accuracy, and recall are very much satisfactory compared to the existing systems.

## 1. Introduction

Diabetes mellitus (DM) is a most important worldwide health concern, which causes a range of long-term complete impairments that have a significant influence on the patient and society, as the illness usually upsets people in their best fruitful ages. As per the latest alarming statistical facts released by the International Diabetes Federation (IDF) Diabetes Atlas 2021 document [1], there are 537 million (1 in 10) adults (aged 20–79 years) living with diabetes mellitus worldwide in 2021. This number is anticipated to rise to 643 million (1 in 9 adults) by 2030 and 784 million (1 in 8 adults) by 2045. Also, 81% (4 in 5 adults) with diabetes are living in middle- and low-income countries. Around 6.7 million deaths (1 every 5 seconds) occurred in 2021 due to DM. An assessed 44% of adults (240 million) living with diabetes in middle and low-income countries are left undiagnosed. In 2021, the global health expenditure caused by diabetes alone is evaluated at USD 996 billion with an increase of 316% over the last 15 years. Around 541 million (1 in 10) adults worldwide have been positioned at high risk of emerging type 2 diabetes due to diminished glucose tolerance levels. The top 10 countries with the maximum number of people with diabetes account for 68% of adults with diabetes [2].

The typical symptoms of type 1 diabetes are frequent urination or bedwetting, constant hunger, excessive thirst, lack of energy or fatigue, blurred vision, sudden weight loss, and diabetic ketoacidosis. Type 2 diabetes is the most common type of diabetes, accounting for over 90% of all diabetes worldwide. Type 2 diabetes also have similar symptoms to type 1 diabetes people but, in general, the status of people may be symptomless and less dramatic. Consequently, one-half of the people suffer from type 2 diabetes due to undiagnosed and continue with prediabetes condition. If this persists for a long duration, the people will develop the health complications such as kidney disease, neuropathy, retinopathy, foot ulceration, peripheral artery disease, heart disease or stroke, very poor healing of lower-limb ulcers, and visual impairment. An effective lifestyle management and consistent health check-ups or screening are a couple of the best precautionary treatments for preventing the development and progress of the mentioned complications [3].

People who have diabetes for a longer duration and poorly controlled blood sugar level, the more prospective they can develop diabetic retinopathy (DR), which causes loss of vision by the gradual destruction of the blood vessels of the retina (a light-sensitive tissue at the back of the eye) over a period of time [4]. The symptoms of DR include blurred vision, difficulty seeing well at night, seeing floaters or spots, and having a dark or empty spot in the centre of the vision. DR is categorized into two types as follows: nonproliferative diabetic retinopathy (NPDR) is the premature period of the disease in which symptoms will be insignificant or nonexistent. In NPDR, tiny blood vessels leak blood and other fluids due to weakness. Fluid might leak into the macula (a retinal tissue liable for clear central vision), which causes the macula to swell, resulting in cloudy or blurred vision. Proliferative diabetic retinopathy (PDR) is the further progressive form of the disease. At this phase, circulation complications deprive the retina of oxygen. As a result, new, fragile blood vessels can begin to grow in the retina and into the vitreous, and the gel-like fluid fills the back of the eye. The new blood vessels may leak blood into the vitreous (centre of eye), causing cloudy vision.

As far as health issues are concerned, the prevention is better than cure. Moreover, the treatment of a specific disease can be easy and effective if it is detected at the premature stage itself. Repetitive medical check-ups play a key role in DR because it exhibits mild symptoms until it is too late for actual treatment [5–7]. If you have either type 1 or type 2 diabetes, your physician may endorse that you have a comprehensive eye assessment straightaway once diabetes is diagnosed. A comprehensive eye scrutiny by an ophthalmologist or optometrist can detect edema (swelling) in the macula at the back of eye. The macula is critical to our central vision, which allows us to see in fine detail. Optical coherence tomography (to check the current status of the retina) and fluorescein angiography (to assess unusual blood vessel growth) are a couple of diagnosis approaches used by physicians through direct fundus examination or fundus photographs. Nevertheless, there is a shortage of knowledgeable ophthalmologists who can assess the fundus photographs to detect DR, which sometimes results in misdiagnosis. Moreover, there is a scarcity of knowledgeable physicians in local areas where there are more diabetic patients and the investigation procedure is onerous. Thus, the need for an automated diagnosis infrastructure for effective time and cost saving instead of manual diagnosis.

Deep learning (DL) is a slice of machine learning techniques, which has been extensively applied for the detection and classification of DR. The DL-based approaches were considered for computer-aided medical diagnosis of DR include support vector machine (SVM), convolutional neural networks (CNNs), restricted Boltzmann machines, sparse coding, and auto encoder [8–10]. All these approaches follow the common procedure, collect the retina image data set, do preprocessing, extract features, and classify using the DL technique. The retinal image data set consists two types of images, i.e., optical coherence tomography (OCT) and fundus colour images [11]. OCT images are either 2 or 3-dimensional, which gives significant evidence about retina thickness and its structure. The fundus images are large 2-dimensional image views of the top layer of retina [12]. The algorithm, which is used for extracting features from the image data set, plays a significant role in the outcome of the experiment. It is better to apply the best optimizer to find the significant features from the data set in the public domain, which might contain some insignificant features also. The principal component analysis (PCA) is the well-established unsupervised machine learning technique for the feature engineering process, which includes extraction and dimensionality reduction [13–15].

In reference [16], the authors proposed a hybrid model for early detection of DR. This model consists of feature selection using PCA, dimensionality reduction using firefly algorithm (FA) [17], and classification using deep learning technique. The FA was the meta-heuristic algorithm and was motivated by the flashing behaviour of fireflies. The algorithm mimics how fireflies interact using their flashing lights. The algorithm assumes that all fireflies are unisex, which means any firefly can be attracted by any other firefly; the attractiveness of a firefly is directly proportional to its brightness, which depends on the objective function. The FA algorithm suffers from trapping itself in the local optimum and has a sluggish convergence speed. So, FA is not a perfect solution to achieve our objective to optimize the dimensionality reduction process during the feature extraction from the image data set. The latest Harris hawks optimization (HHO) algorithm resolves hitches of a feature space comprising multi-modality, local optimal solutions, and misleading optima [18]. HHO is a population-based metaheuristic algorithm, inspired by the hunting strategy and cooperative behaviour of Harris hawks. As far as our knowledge, HHO does not apply so far for DR detection and classification during the feature engineering process. The main objectives of this research work are optimizing the feature set extracted from the image data set and proposing the best classification algorithm through experiments. The contributions of this work are as follows:

- A principal component analysis algorithm has been used for feature extraction and selection from the image data set.
- A dimensionality reduction using the HHO algorithm has been proposed in order to optimize the feature set further.
- A deep convolution neural network has been used for DR detection and classification.
- The proposed work, a combination of deep neural network, PCA, and HHO, has been implemented and compared with various machine learning models such as KNN (k-nearest neighbour), SVM (support vector machine), and XGBOOT classification algorithm.
- The numerical outcomes of this experiment along with comparisons are encouraging and are better than entrenched metaheuristic methods in terms of accuracy, precision, recall, specificity, and sensitivity.

The subsequent sections of this study are organized as follows: a noticeable literature review has been presented in Section 2.  Section 3 deliberates the proposed methodology and experimental setting details.  Section 4 discusses the results achieved with the proposed method and compared with existing approaches and Section 5 articulates the conclusion and scope of future work.

## 2. Literature Review

Today's world of advances in deep learning (DL) has changed the way, in which healthcare is handled recently, which allows the medical practitioners effectively diagnose and treat diseases. Several researchers across the globe attempted to address the task at hand effectively. From the evolution of various DL-based classification, detection models have been drawn in this century. Many researchers are working in this area across the globe.  Table 1 lists the review on deep learning applications in diabetic retinopathy and other datasets.

Currently, DL has begun to have an immense impact in various fields of health care. The rapid development of variations in DL techniques and the increased availability of data in health care have allowed the recording of impressive health care results [26, 27]. DL approaches can uncover details contained in a vast volume of health care data that are clinically important, which can be used for treatment, monitoring, prevention, and decision-making of health conditions. EHR processing, health behaviour reaction, and sound treatment retrieval from eye-related research, text, and classification are some of the implementation areas of DL. This will lead to simpler treatment for patients, with quicker and more effective monitoring. Usage of DL in medicine has converted the use of basic instruments, such as stethoscopes and thermometers, into computed tomography (CT), lithotripsy, ventilators, radio nuclear imaging, radiation therapy, ultrasound diagnostic devices, and dialysis, which has been used for highly adaptive treatment for traditional medical care capable of dealing with many dreaded illnesses. There is no question that health care treatment and facilities will see greater changes in even other sectors in the coming years to make them more competitive with qualitative programs.

Many international collaborative works have focused on applying DL-based algorithms for the diagnosis of diabetic retinopathy disease. The authors in reference [28] explored DL applications for a range of biomedical issues, including simple biological processes, patient classification, and patient care, and addressed if the DL can transform the mentioned activities or if the biomedical sphere faces special challenges. They notice that DL is yet to definitively overcome or revolutionize biomedicine in any of the important problems in the field after a comprehensive literature review, but promising progress is made on the previous significant works. While changes over previous works have been typically small, recent developments suggest that deep learning approaches can offer useful means to speed up or sustain human science. While progress has been made in connecting the prediction of a particular NN to input features, it remains an open challenge to understand how users can view these models to generate testable predictions of the system under research. However, DL is expected to show promising results in biological applications.

In the last few decades, diabetic retinopathy become a global medical problem among elderly people. The authors in reference [29] have explored DNN to predict diabetic retinopathy. They have proposed a principal component analysis (PCA)-based DNN model for the classification. The grey wolf optimization (GWO) algorithm is used to extract features of diabetic retinopathy dataset. The proposed model has compared their prominent results with pre-existing techniques like XGBoost, k-NN, Naive Bayes, and support vector machine (SVM). Reference [30] presented an approach for multimodal fusion in the contourlet domain based on weighted PCA. The main purpose of using contourlet transform is because of capability to capture visual geometrical anisotropy and structures. Further, weighted PCA minimizes the dimensionality of the source images and improves better selection of principal components.

Maximum and minimum fusion methods are used to fuse the decomposed coefficients. Image quality is assessed quantitatively using conventional fusion metrics to evaluate the fused image in terms of both information content and reconstruction quality. Reference [31] extracted the optimal features from the heart disease dataset through the proposed dimensionality reduction technique. The dataset used in this work was obtained from the heart disease dataset from the publicly available UCI machine learning repository. This dataset has 74 features. They have used 6 ML classifiers to validate the proposed model. Random forest (RF) integrated with chi-square-based PCA (CHIPCA) yielded the highest accuracy of 99.4% for Cleveland Hungarian (CH), 99.0% for Hungarian, and 98.7% for Cleveland datasets.

Reference [32] developed a model based on each patient's risk factors. For each stage of DR advancement, the author proposes a model to estimate the time and rate of progression. The proposed model could aid physicians in creating a customised follow-up program for patients depending on their disease stage and risk factors. Reference [33] attempted to optimize the energy utilization in the IoT networks through an optimal CH selection using a nature-inspired algorithm, HHO. The performance of the HHO-based CH model was analysed through several parameters such as load, Temperature, number of alive nodes, delay, and residual energy.

The authors in reference [34] used a model to process the images in order to distinguish the ocular structure and detect the existence of diabetic retinopathy in this study. For mapping an image with the relevant label, the model's parameters were optimized using the transfer-learning process. The author has used the medical fundus oculi images dataset for training and testing a model. The proposed study has a 97.78% accuracy rate for the accurate prediction of diabetic retinopathy in fundus oculi images. Many authors in reference [35] applied this dataset to DNN and CNN.

For these kinds of applications, image data alone is not sufficient. Many works have been done in this area but they did not find out a prominent method or model to improve the detection of diabetic retinopathy.

## 3. Proposed Method

### 3.1. Harris Hawks Optimization (HHO)

In this section, we describe the mathematical model of the proposed method along with its usage for optimal results. In general, population-based metaheuristic optimization algorithms mimic the natural concept by considering a set of solutions (populations) during the optimization phase of each iteration [36]. The latest such algorithm, the Harris hawks optimization (HHO) technique, is a gradient-free, metaheuristic, swarm-based, nature-inspired algorithm [18]. The key concept of this algorithm is the utilization of dynamic and natural cooperative hunting behaviour of Harris' hawks for victims (medium-sized preys such as rabbits, hares, reptiles, ground squirrels, quail, birds, and other rodents) [37]. The hunting patterns exhibit the Harris' hawk's intelligent behaviour despite the complex dynamic environment and escaping nature (zig-zag gestures) by victims. So, an optimization technique, which simulates the behaviour of this hunting pattern, can give better results compared to the existing techniques. The main advantages of the HHO algorithm are the possibility of getting a global optimal solution, high convergence speed, high accuracy, and better quality. Consequently, the HHO algorithm can be applied to solve various optimization problems in the engineering domain such as feature extraction, design and development of a model, pattern recognition, and electrical and electronics optimal design applications [33].

**Figure 1:**
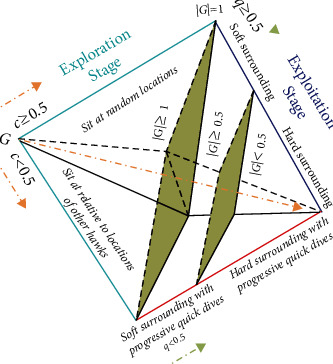
Various stages during the HHO algorithm.

The working mechanism of the HHO algorithm in different stages is depicted in Figure 1 and the next subsections follow its description in a stage-by-stage manner. Broadly, the algorithm consists of two stages: exploration and exploitation. The exploration stage models the behaviour of the Harris hawks search process to detect and spot the victims. The exploitation stage models the intelligent hunt.

#### 3.1.1. Exploration Stage

Usually, the hawks who have powerful eyes will spend hours of time patiently to track and detect the victims by waiting, observing, and monitoring the desert spot. In the context of HHO for optimization purposes, the hawks are observed as candidate solutions and the best of the candidate solutions at each iteration process are observed as intended optimal solution or victim. Initially, the hawks can perch at location of the spot in two possible ways, either close enough relative to the location of the other family hawks and the victim or at random locations on high trees. Both the ways are considered with an equal chance of probability and the same is modelled in equation (1) with *c* ≥ 0.5 for random locations and *c* < 0.5 for relative locations. Several hawks cooperatively move towards the victim from different directions to surprise it. The change in the location of the hawks at each iteration during the exploration stage is mathematically modelled as follows:

(1) $$\begin{array}{c} C\left(i+1\right)=\left\{\begin{array}{c} C_{\text{random}}\left(i\right)-n_{1}\left|C_{\text{random}}\left(i\right)-2n_{2}C\left(i\right)\right|\\ c\geq 0.5\\ \left(C_{\text{victim}}\left(i\right)-C_{avg}\left(i\right)\right)-n_{3}\left(L+n_{4}\left(U-L\right)\right)\\ c<0.5 \end{array}\right., \end{array}$$

where *C*(*i*) is the current location vector of hawks. *C*(*i* + 1) is the location vector of hawks in the next iteration *i*. *C*_victim_ (*i*) is the location of victim. *C*_avg_ is the average location of the current population of hawks. The *c*, *n*_1_, *n*_2_, *n*_3_, and *n*_4_ are random variables whose values are to be updated at each iteration between 0 and 1. The upper and lower bounds of these variables are considered as *U* and *L*, respectively. The randomly picked hawk from the current population is represented by *C*_random_(*i*). The average location of the hawks is calculated using equation (2):

(2) $$\begin{array}{c} C_{\text{avg}}\left(i\right)=\frac{1}{M}\sum_{k=1}^{M}C_{k}\left(i\right), \end{array}$$

where *C*_*k*_(*i*) denotes the position of hawks after iteration *i* and *M* represents the number of hawks.

#### 3.1.2. Exploitation Stage

In this stage, the hawks execute the sudden pounce on the envisioned victim, which was spotted in the exploration stage. In this context, several styles of chasing will take place due to the victims may execute different escaping strategies according to the dynamic environment. In the HHO, four potential tactics were provided based on chasing styles of the hawks and escaping attitudes of the victim. Let *q* represents escaping chance of a victim from sudden pounce, successful escape with *q* < 0.5, and unsuccessful escaping with *q* ≥ 0.5. However, the hawks have their own strategies such as soft or hard surround to catch the victim. In other words, the retained energy of the victim gives directions to the hawks to do either soft or hard encircle the victim. To win this hunting process, the hawks will try to reach closer and closer to the envisioned victim and then cooperatively executes the sudden pounce to kill the same. Simultaneously, the victim looses its energy while using escaping strategies. After some time, the victim energy will be exhausted and this context leads to catch the same easily by hawks.

The modelling of the victim energy plays a vital role here and it is defined as follows:

(3) $$\begin{array}{c} G=2G_{0}\left(1-\frac{i}{I}\right), \end{array}$$

where *G* represents the victim escaping energy. *I* is the maximum possible iterations. *G*_0_ is the initial energy state and it is a random variable whose value changes between −1 and 1 in each iteration. The victim is strengthening if the value of *G*_0_ increases from 0 to 1 and weakening if the value of *G*_0_ decreases from 0 to −1. However, the dynamic value of escaping energy *G* is always in the downtrend during iteration by iteration. The HHO moves to the exploration stage to search for another victim if |G| ≥ 1 and continues in the exploitation stage if |G| < 1. During the exploitation stage, the algorithm switches between soft (if |G| ≥ 0.5) and hard (if |G| < 0.5) surrounding of the victim. So, the hawks will execute any one of the following four promising tactics based on the *G*, the escaping energy of the victim, and *q*, the escaping chance of a victim.

#### 3.1.3. Soft Surrounding

This scenario is applicable, if |G| ≥ 0.5 and *q* ≥ 0.5. Here, the victim tries to escape from hawks through confusing zig-zag movements/jumps with having sufficient energy. At this juncture, the hawks follow a soft surrounding approach to do sudden pounce in several rounds of attempts by making the victim energy exhausted. This soft surrounding approach has been modelled as follows:

(4) $$\begin{array}{c} C\left(i+1\right)=\Delta C\left(i\right)-G\left|SC_{\text{victim}}\left(i\right)-C\left(i\right)\right|. \end{array}$$

(5) $$\begin{array}{c} \Delta C\left(i\right)=C_{\text{victim}}\left(i\right)-C\left(i\right), \end{array}$$

where ∆C(*i*) is the difference between the location vector of the victim and the present location in iteration *i*. The strength of the victim to do zig-zag movement or jump is represented by *S* = 2(1 − n5). Here, *S* value updates dynamically in each iteration to mimic the movements or jumps by victims. N5 is a random variable whose value is between 0 and 1.

#### 3.1.4. Hard Surrounding

**Figure 2:**
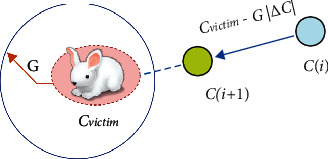
Illustration of overall vectors in the strategy of hard surrounding with one hawk.

This scenario is applicable, if |G| *<* 0.5 and *q* ≥ 0.5. Here, the victim energy is so exhausted and its escaping energy is low. At this juncture, the hawks follow a hard surrounding approach to do sudden pounce to catch the intended victim. Here, the current locations of the hawks are updated using equation (6) and this concept with one hawk is illustrated in Figure 2:

(6) $$\begin{array}{c} C\left(i+1\right)=C_{\text{victim}}\left(i\right)-G\left|\Delta C\left(i\right)\right|. \end{array}$$

#### 3.1.5. Soft Surrounding with Progressive Quick Dives

This scenario is applicable, if |G| ≥ 0.5 and *q* < 0.5. This means, the hawks construct a more intelligent soft surrounding strategy due to the victim still has sufficient energy to escape successfully [37]. In real time, the victim chooses random escaping patterns and leapfrog movements [38]. To model this concept, the levy flight (LF) random walk notion is adopted in to the HHO algorithm [39,40]. The LF concept helps to mimic the real-time zig-zag movements of victim and the hawks' sudden, irregular, and quick dives around the escaping victim. The hawks dynamically execute many sudden quick dives around the victim, and update their location and direction of attack progressively according to the escaping behaviour of victim. The literature proved that the LF-based actions are the optimal searching strategies for hunters in nondestructive searching circumstances. Moreover, the LF-based movement of patterns is common in the victims such as rabbit, monkeys, and sharks. This motives the utilization of LF-based movements within the HHO algorithm.

To catch the victim in the adverse condition, the hawks' real behaviour is to choose the best possible dive at every step and reach closer by closer to the victim. To mimic this behaviour, the hawks can compute their next movement based on the following rule:

(7) $$\begin{array}{c} A=C_{\text{victim}}\left(i\right)-G\left|SC_{\text{victim}}\left(i\right)-C\left(i\right)\right|. \end{array}$$

After computing the next move, the hawks compare the latest move with the previous one. If the latest is better than the previous one, then they choose and execute the same. Otherwise, they choose sudden, irregular, and quick dives around the escaping victim. Sometimes, the hawks choose to dive according to LF-based patterns as defined in the following equation:

(8) $$\begin{array}{c} B=A+V*LF\left(D\right), \end{array}$$

where *D* is the problem dimension, *V* is a random 1Xd dimensional vector, and the levy flight (LF) function is computed using the following equation:

(9) $$\begin{array}{c} LF\left(x\right)=0.01\times \frac{p\times \sigma }{{\left|q\right|}^{1/\beta }},\sigma ={\left(\frac{\Gamma \left(1+\beta \right)\times \text{sin}\left(\pi \beta /2\right)}{\Gamma \left(1+\beta /2\right)\times \beta \times 2^{\left(\beta -1/2\right)}}\right)}^{1/\beta }, \end{array}$$

where *β* is a constant with a value of 1.5 and *p* and *q* are random variables with a value between 0 and 1.

**Figure 3:**
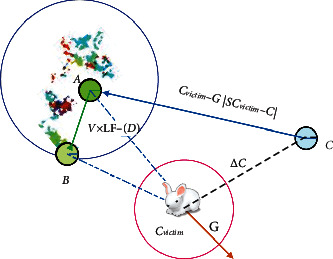
Illustration of overall vectors in the strategy of soft surrounding with progressive quick dives.

Therefore, in the soft surrounding phase, the latest positions of the hawks can be defined in the final strategy using the following equation:

(10) $$\begin{array}{c} C\left(i+1\right)=\left\{\begin{array}{cc} A & if\;F\left(A\right)<F\left(C\left(i\right)\right)\\ B & if\;F\left(B\right)<F\left(C\left(i\right)\right) \end{array}\right., \end{array}$$

where A and B are attained using equations (7) and (8), respectively. An instance of the above modelling concept for one hawk is illustrated in Figure 3. This demonstration also contains the leapfrog movements based on LF through possible iterations. The LF-based patterns are depicted with coloured dots on trial and then the HHO algorithm touches position B. In every stage, the next better location is chosen as A or B. The same concept is applicable to all hawks during searching.

#### 3.1.6. Hard Surrounding with Progressive Quick Dives

**Figure 4:**
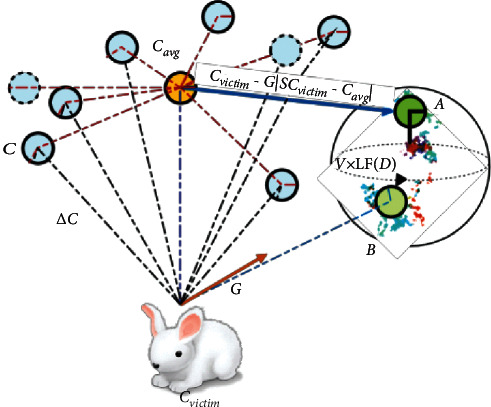
Illustration of overall vectors in the strategy of hard surrounding with progressive quick dives.

This scenario is applicable, if |G| *<* 0.5 and *q* < 0.5. This means, the hawks execute hard surrounding before the sudden pounce to catch and kill the victim due to the victim energy has been exhausted. At victim side, this condition is similar to the soft surrounding, but the hawks move closer by closer by reducing the average position with the escaping victim. Hence, the hawks update their locations in the context of hard surrounding using the following equation:

(11) $$\begin{array}{c} C\left(i+1\right)=\left\{\begin{array}{cc} A & if\;F\left(A\right)<F\left(C\left(i\right)\right)\\ B & if\;F\left(B\right)<F\left(C\left(i\right)\right) \end{array}\right., \end{array}$$

where A and B are attained using equations (12) and (13), respectively:

(12) $$\begin{array}{c} A=C_{\text{victim}}\left(i\right)-G\left|SC_{\text{victim}}\left(i\right)-C_{\text{avg}}\left(i\right)\right|. \end{array}$$

(13) $$\begin{array}{c} B=A+V\times LF\left(D\right), \end{array}$$

where *C*_*avg*_(*i*) is computed using equation (2). An instance of the above modelling concept with overall vectors for one hawk is illustrated in Figure 4. This demonstration also contains the leapfrog movements based on LF through some iterations. The LF-based patterns are depicted with coloured dots and the next better location is provided by A or B for the next iteration.

The key feature of HHO algorithm, which has a productive impact during the exploitation stage, is that it exhibits a series of search strategies and picks the best move at each iteration. The HHO algorithm tries to improve the superiority of the solution throughout the optimization process by progressive choice arrangement by search agents. The usage of adaptive and time-varying constituents allows the HHO algorithm to resolve the hitches of a feature space comprising multimodality, local optimal solutions, and misleading optima. The candidate solutions take help from the strength of randomized moves in congruent during the stages of exploration and exploitation learning.

Finally, the computational complexity of the HHO algorithm has been analysed and computed based on three tasks: initialization, fitness evaluation, and updating of hawks. If there are N number of hawks, then the computational complexity of the initialization process is O(N). If *D* is the dimension of definite problem and *M* is the maximum number of iterations then the computational complexity of the updating procedure is O(*M*  ×  *N*) + O(*M*  ×  *N*  ×  D), which is a sum of searching for the best location and updating the location vector of all hawks. Therefore, the computational complexity of HHO is O(*N*  ×  (*M* + MD +1)).

## 4. Results and Explanation

The dataset, the experimental framework, the metrics, and the experimental outcomes are all discussed in this section. There were 1151 instances and 20 attributes in the diabetic retinopathy dataset used in this study.  Table 1 [16] lists the characteristics of the dataset used in this study. Except for the output layer, all of the layers used the Softsign activation function. The experiment was conducted using the Diabetic Retinopathy Debrecen dataset [41] from the UCI ML library. The features extracted from the image dataset were used to create the attributes in this dataset. The Python experimentation was carried out on a personal machine with 8 GB of RAM. The results of the current proposed work are outperformed than the existing methodologies discussed in the literature review.

### 4.1. Metrics Used in the Evaluation of the Model

The suggested model is evaluated using the metrics listed below.

*Accuracy*. In the testing phase, it is the percentage of proper predictions made by a classifier and the actual value of the label. It is also known as the ratio of the number of right assessments to the total number of assessments. The following equation can be used to calculate accuracy:

(14) $$\begin{array}{c} \text{Accuracy}=\frac{\left(TNeg+TPos\right)}{\left(TNeg+TPos+FNeg+FPos\right)}. \end{array}$$

Here, TPos is true positives, TNeg is true negatives, FPos is false positives, and FNeg is false negatives.

When the class label of a record is available in the given dataset and the classifier indicates positive for that record, then it is called a true positive. When the class label of a record is not available and the classifier forecasts the class label, then it is called a true negative. When the class label of a record is accessible and the classifier expects a negative for that record, then it is called a false negative. When the class label of a record is not available in a dataset and the classifier estimates a positive class, then it is called a false positive.

*Specificity*. It is the percentage of true negatives successfully detected by the classifier while taking the test. The following equation is used to calculate it:

(15) $$\begin{array}{c} \text{Specificity}=\frac{\left(Tneg\right)}{\left(Tneg+Fpos\right)}. \end{array}$$

*Sensitivity*. During testing, it is the percentage of true positives successfully detected by the classifier. The following equation is used to calculate it:

(16) $$\begin{array}{c} \text{Sensitivity}=\frac{\left(TPos\right)}{\left(TPos+FNeg\right)}. \end{array}$$

*Precision*. Precision is an important metric for measuring exactness. It expresses how much of the total forecasted positive occurrences the classifier identified as positive, as shown in the following equation:

(17) $$\begin{array}{c} \text{Precision}=\frac{\left(Tpos\right)}{\left(Tpos+Fpos\right)}. \end{array}$$

*Recall*. The percentage of positive cases classified as positive by the classifier is determined by recall. When there is a large cost connected with the false negative, as stated in equation (18), the recall is a performance parameter used to predict the optimal model:

(18) $$\begin{array}{c} \text{Recall}=\frac{\left(Tpos\right)}{\left(Tpos+Fneg\right)}. \end{array}$$

### 4.2. Performance Analysis

**Figure 5:**
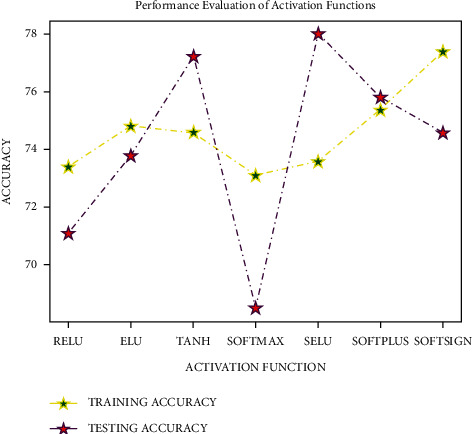
Analysis of activation functions.

The DNN-PCA model was built using a sequential strategy for testing the proposed model. The dataset was divided into two sections for cross validation, with 80% utilised for training and 20% used for verifying every 64 records (batch size). To determine the optimal activation function for a dataset of 50 epochs and a batch size of 64, testing was performed on many activation functions such as ReLU, ELU, tanh, Softmax, SELU, Softplus, and Softsign. The Softsign activation function, as shown in Figure 5, provided the best average training and testing accuracy. As a result, the Softmax activation function is used to evaluate the model on dense layers.

**Figure 6:**
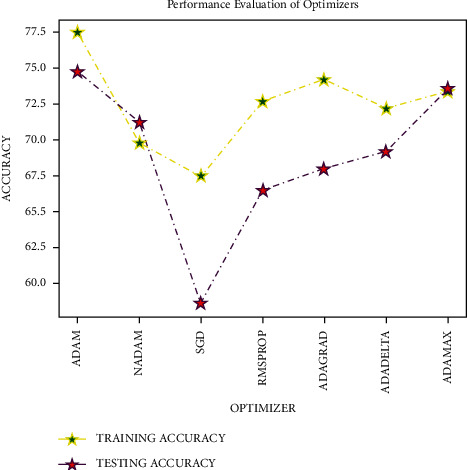
Analysis of optimizers.

The investigation was completed on the dataset with 50 epochs and a batch size of 64 to obtain the optimal optimizer in the layers of deep neural networks using many optimizers such as Adam, NAdam, SGD, rmsprop, adagrad, adadelta, and adamax. As shown in Figure 6, the Adam optimizer offered the highest level of accuracy. As a result, the Adam optimizer is selected for input and other dense layers experimentation. For the output layer, a sigmoid optimizer is employed.

**Figure 7:**
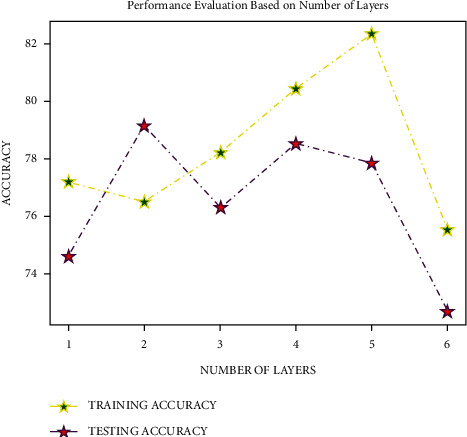
Analysis based on the number of layers.

The DR dataset was explored with many layers with 50 epochs, Adam optimizer at input and dense layers, Softsign activation function, sigmoid optimizer at the output layer, and 64 as batch size to pick the number of layers in DNN for experimenting. The model showed an excellent training and testing accuracy with 5 layers, as shown in Figure 7, with the accuracy level starting to decrease with 6 levels. As a result, a five-layer deep neural network was deployed in the experiment.

**Figure 8:**
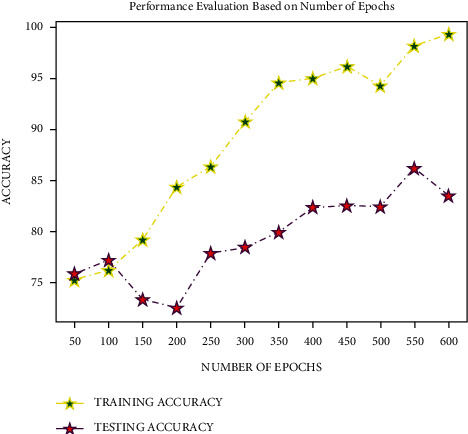
Analysis based on the number of epochs.

The DR data set was tested utilising Softsign activation with five intermediate layers, sigmoid optimizer at the output layer, Adam optimizer at input and dense layers, and 64 as batch size to determine the number of epochs. The model provided better results in providing a good average training and testing accuracy with 600 epochs, as shown in Figure 8, with the testing accuracy beginning to dip with 650 epochs. As a result, 600 epochs were used to train a deep neural network.

The number of components chosen for the PCA in the experimental study was 0.9%, that is, to retain 99% of the information

**Figure 9:**
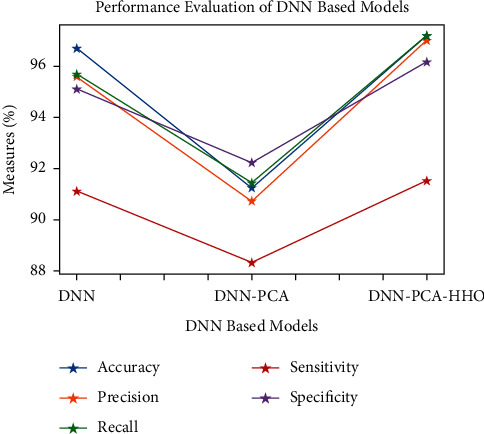
Analysis of DNN-based models.

**Figure 10:**
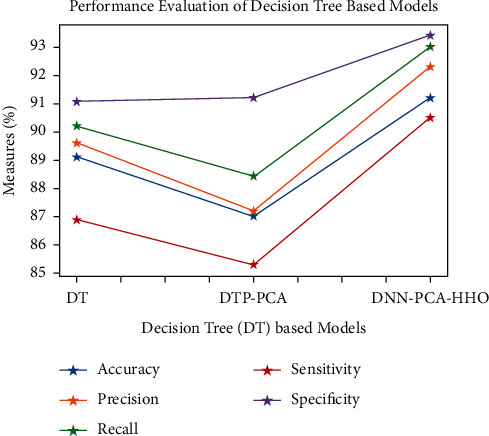
Analysis of DT-based models.

**Figure 11:**
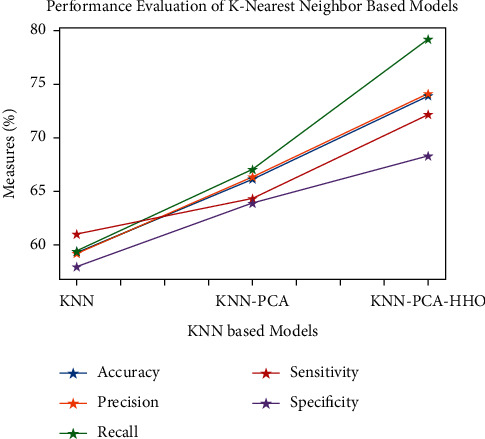
Analysis of KNN-based models.

**Figure 12:**
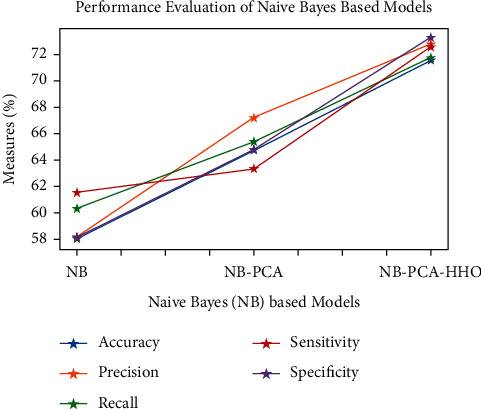
Analysis of NB-based models.

**Figure 13:**
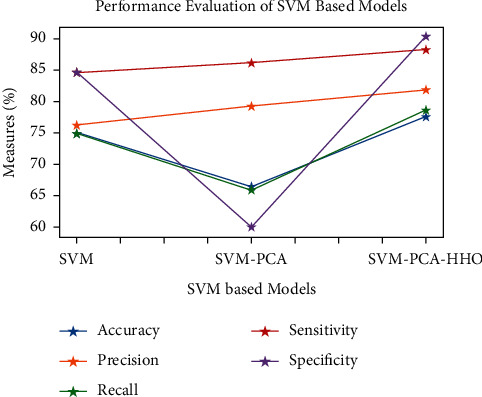
Analysis of SVM-based models.

**Figure 14:**
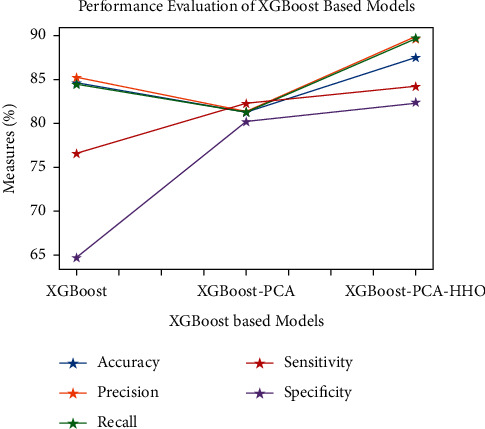
Analysis of XGBoost-based models.

Figures 9–14 show how the accuracy, precision, recall, sensitivity, and specificity of machine learning models are evaluated. These graphs show that ML models based on PCA-HHO provided the best results rather than the other two scenarios: ML with PCA and ML without dimensionality reduction. When dimensionality reduction and feature engineering concepts are included or excluded from ML methods, it is found that the suggested model, DNN-PCA-HHO, outperforms the other hybrid ML techniques.

**Figure 15:**
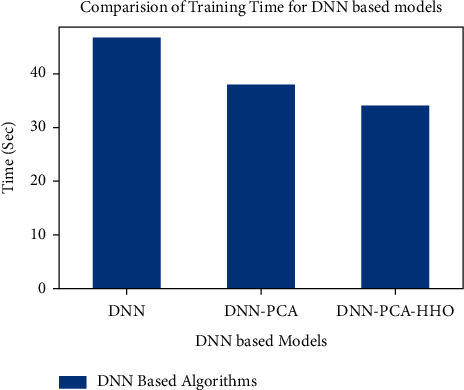
Training time analysis for DNN-based models.

The following are the highlights of the results relevant to the suggested model:

1. When compared to other prominent ML hybrid models, the DNN-PCA-HHO model surpasses them.
2. When PCA is used alone on DNN and other ML algorithms, the performance measurements deteriorate slightly. However, the amount of time spent training is minimised.
3. PCA + HHO, on the other hand, improves the performance of ML algorithms while cutting training time in half, as shown in Figure 15.
4. When the original dataset was employed, it fell victim to overfitting, which had a negative impact on the results. The performance has been improved even though the size of the data set was increased by two times and the same is shown in the Figure 13.

Table 2 describes the findings of the analysis.

## 5. Conclusion

The proposed system used principal component analysis for extracting the best features. The dataset for the proposed model is gathered from the publicly accessible UCI machine learning repository, which contained redundant and unnecessary features in its raw form. The Harris hawks optimization algorithm outperformed the process of selection and extraction of the required features from the dataset. The proposed model results were compared to the outcomes of the most popular machine learning algorithms, with the findings demonstrating the model's superiority in terms of specificity, precision, accuracy, recall, and sensitivity. Nevertheless, in the event of a low-dimensional dataset, the model's ability to perform well may be limited by the possibility of overfitting. Therefore, our proposed model encourages the researchers to pursue similar research in a variety of other health disciplines using high-dimensional data.

## Figures and Tables

**Algorithm 1:**
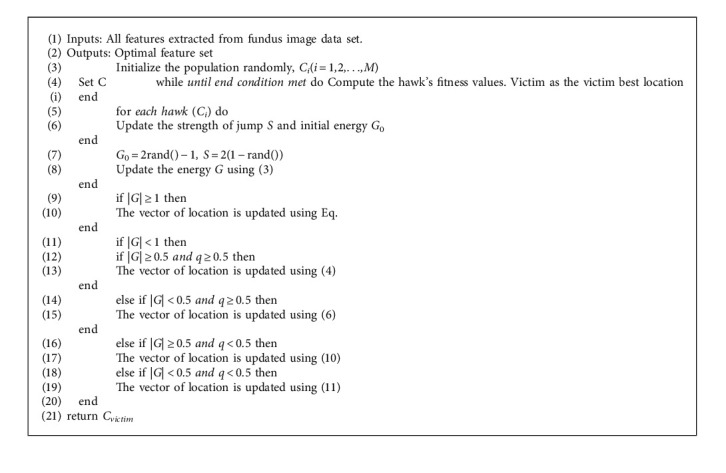
HHO Algorithm Pseudocode [18].

**Table 1:** Review of deep learning applications in diabetic retinopathy and other datasets.

Reference	Dataset	Method used	Evaluation metrics	Research challenges
[19]	Diabetic retinopathy (DR) dataset consisted of 75137 images	5-Fold cross-validation and data-driven deep learning algorithm	Sensitivity, specificity, and AUC score	The results were not properly evaluated using typical state-of-the-art models
[20]	73 patients (122 eyes) were evaluated, 50.7% men and 49.3% women	RBM-1000, RBM-500, and OPF-1000	Sensitivity measured, specificity, and accuracy	More in-depth analysis on larger datasets was missing and accuracy may also be improved
[21]	14,186 retinal images and Messidor dataset with 1200 images	Deep learning algorithm	Accuracy, sensitivity, specificity, positive and negative predictive values, and AUC	Dataset is fixed and is not compared with other technique
[22]	128175 retinal images, EyePACS-1 dataset consisted of 9963 images, and Messidor-2 dataset with 1748 images	Deep convolutional neural network	The algorithm had 97.5% and 96.1% sensitivity and 93.4% and 93.9% specificity in the 2 validation sets	Limited dataset, system maybe failed to learn more complex features
[23]	Heart disease dataset	Effective heart disease prediction system using enhanced deep genetic algorithm and adaptive Harris hawks optimization-based clustering	Accuracy, precision, recall, specificity, and F-score	Requires more improvement in the learning process
[24]	COVID-CT-dataset: 349 and 397 images and CT scans for COVID-19 classification: 4,001 and 9,979 images	Hybrid learning and optimization approach CovH2SD-CovH2SD uses DL. HHO algorithm to optimize the hyperparameters	Accuracy, precision, recall, F1-score, and AUC performance metrics	Not good for multiclass classification
[25]	Hand gesture dataset from Kaggle repository	HHO is used for hyperparameter tuning of CNN for enhancing hand gesture recognition	Reduction of the burden on the CNN by reducing the training time and 100% accuracy for hand gesture classification is attained	Requires more improvement in the learning process

**Table 2:** Summary of the experimental results.

Metric ⟶method ↓	DNN	DNN-PCA	DNN-PCA-HHO	DT	DT-PCA	DT-PCA-HHO	KNN	KNN-PCA	KNN-PCA-HHO	NB	NB-PCA	NB-PCA-HHO	SVM	SVM-PCA	SVM-PCA-HHO	XGBoost	XGBoost-PCA	XGBoost-PCA-HHO
Accuracy	96.7	91.2	97	89.1	87	91.2	59.4	66	73.9	58	65	71.5	75	66	77.5	84.5	81	88
Precision	95.6	90.7	97	89.6	87	92.3	59.2	66	74.1	58	67	72.7	76	79	81.7	85.2	81	90
Recall	95.7	91.4	97	90.2	88	93.0	59.3	67	79.1	60	65	71.6	75	66	78.6	84.3	81	90
Sensitivity	91.1	88.3	91	86.9	85	90.5	60.9	64	72.1	61	63	72.5	84	86	88.2	76.5	82	84
Specificity	95.1	92.2	96	91.1	91	93.4	57.9	64	68.2	58	65	73.2	85	60	90.3	64.6	80	82

## Data Availability

The data can be provided based on the request from the corresponding author.
